# Network motifs modulate druggability of cellular targets

**DOI:** 10.1038/srep36626

**Published:** 2016-11-08

**Authors:** Fan Wu, Cong Ma, Cheemeng Tan

**Affiliations:** 1 Department of Biomedical Engineering, University of California, Davis, 1 Shields Ave, Davis, CA 95616, USA; 2 Computational Biology Department, School of Computer Science, Carnegie Mellon University, 5000 Forbes Ave., Pittsburgh, PA 15213, USA

## Abstract

Druggability refers to the capacity of a cellular target to be modulated by a small-molecule drug. To date, druggability is mainly studied by focusing on direct binding interactions between a drug and its target. However, druggability is impacted by cellular networks connected to a drug target. Here, we use computational approaches to reveal basic principles of network motifs that modulate druggability. Through quantitative analysis, we find that inhibiting self-positive feedback loop is a more robust and effective treatment strategy than inhibiting other regulations, and adding direct regulations to a drug-target generally reduces its druggability. The findings are explained through analytical solution of the motifs. Furthermore, we find that a consensus topology of highly druggable motifs consists of a negative feedback loop without any positive feedback loops, and consensus motifs with low druggability have multiple positive direct regulations and positive feedback loops. Based on the discovered principles, we predict potential genetic targets in *Escherichia coli* that have either high or low druggability based on their network context. Our work establishes the foundation toward identifying and predicting druggable targets based on their network topology.

Pharmaceutical industry has invested heavily in the strategy of identifying compounds with high affinity and specificity to single cellular targets. This one-drug-one-target strategy is thought to introduce high effectiveness and low side-effect of drug molecules. However, the strategy is challenged by the continuous high attrition rates of drug molecules in late phases of drug development^1^,^2^,^3^. Furthermore, the strategy contradicts existing understanding that many diseases, such as cancer, diabetes and inflammatory disorders involve multiple pathways and cellular networks. The complexity of cellular networks implies that the paradigm of one-drug-one-target may not be sufficiently predictive for effective treatment^4^. To this end, an alternative paradigm named network (or system) pharmacology has been proposed in drug development^5^,^6^. Network pharmacology investigates cellular targets of drugs by studying the connected networks, including genetic regulatory networks, metabolic networks, and protein-protein interactions. For instance, a network pharmacology approach has proposed that intrinsic robustness of cellular networks against external perturbations is a key factor that produces unexpected inefficiency of many potential drugs^7^. A general consensus from these studies suggests that modulation of multiple targets and understanding of disease related networks will be helpful for effective treatment and next-generation drug discovery.

Previous work in the field of network pharmacology can be generally classified into two areas: multi-target drugs (i.e. polypharmacology) and disease network motifs. Multi-target drugs are designed based on the rationale that complex diseases often involve abnormal functions of multiple cellular components, hence requiring complex drug targeting. Along this line, computational approaches have been used to predict previously unknown molecular targets of commercially available drugs^8^,^9^. Multiple drug molecules have been used in combination to treat certain complex diseases, including dyslipidemia, asthma, and HIV^10^,^11^,^12^. Indeed, these studies have provided insights into side-effects of drugs, new use of existing drugs, and combinational therapy. Another area in network pharmacology investigates disease network motifs and their effects on drug treatment. The advancements in ‘omics’ have enabled the reconstruction of detailed disease-related networks^13^,^14^. Various computational models have also been constructed to develop novel treatment strategies^15^,^16^,^17^. Multiple drug targets are identified based on disease-related molecular networks^15^. Metabolic networks of human are compared with the networks of other species based on structural and functional similarity. The cross-species molecular network association (CSMNA) is used to identify natural products from other species that may be used to treat human diseases^17^. These works have suggested potential drug targets using specific networks assembled from literature data, but they do not lead to fundamental understanding of network structures that modulate inhibition of a single target by drug molecules. A critical knowledge gap exists between the paradigms of one-drug-one-target and network pharmacology, in which it is unclear if unsatisfactory treatment outcomes are due to fundamental features of cellular networks that regulate the target. Can we unveil fundamental principles of cellular network structures to guide our selection of a drug target? Answers to the question will lead to general principles to predict and improve druggability of a cellular target.

Here, we use mathematical and computational approaches to study the characteristics of cellular network motifs and their effects on druggability. We augment the definition of druggability by the likelihood of cellular targets that are embedded in network motifs to be inhibited by drug molecules^18^,^19^. We ask two specific questions: Does a network motif that regulates a cellular target affect its druggability? Can we use basic features of the network motifs to predict and improve its druggability? To answer the questions, we studied all possible three-node motifs to simulate cellular networks, using one of the nodes to simulate a drug target. We quantitatively defined druggability as the reduction of the target concentration after drug inhibition. Through our mathematical analysis, we revealed basic principles of network motifs that modulate druggability and potential ways to improve druggability by selectively perturbing network motifs. We also applied the principles to the genetic network of *E. coli* and identified potential druggable targets.

## Results

### Construct mathematical models to quantify druggability of network motifs

To investigate druggability, we used three-node motifs following a previous work^20^. Each network motif consisted of nodes A, B, and C, as well as nine possible regulatory links between the nodes. Specifically, we simulated node A as the drug target and nodes B and C as buffer nodes that regulate the target. The regulations between nodes can be positive, negative, or null. We setup the model based on three reasons. First, three-node motifs allow comprehensive motif analysis with reasonable computational power. Second, despite the complexity of natural pathways, certain biological components may function similarly and can be organized into network motifs^21^. Third, three-node motifs are the minimal networks that generate all possible fundamental motifs, including positive/negative feedback loops, coherent/incoherent feedforward loops, and network cascades^22^.

We chose Michaelis-Menten kinetics to describe each regulation because they are commonly used to simulate cellular reactions^23^. A drug was simulated by an input that inhibited node A and was changed from 0.1 (I_1_) to 1.0 (I_2_). The concentrations of the input do not affect our conclusions, as long as they increase from a low level to a high level (Supplementary Table S1). For each motif, the simulation was conducted using 1000 sets of randomized parameters. To identify expected druggability of a network motif within the defined parameter range, we averaged druggability from the randomized parameter sets. This calculation leads to conclusions that are consistent with our steady-state analysis, which is independent from the choice of specific parameter sets. Furthermore, we have performed another set of simulations using 100 randomized parameter sets for each motif, and all conclusions remain unchanged (Supplementary Figures S1,S2). Therefore, our conclusions are not sensitive to the size of parameter set. Two states of the system (A_1_ and A_2_) that corresponded to I_1_ and I_2_ were used to evaluate the druggability of each network motif as 
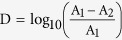
. This metric represents the fractional reduction of the target A in logarithm scale within simulation time (t = 10). A high value of the druggability metric indicates high reduction of A after the addition of a drug, which is considered as a desirable outcome in this study. We note that our conclusions are supported by steady-state analysis. Therefore, the choice of a terminal simulation time will not affect our conclusions (See Supplementary Information Text for steady-state analysis).

### Reveal fundamental features of network motifs that exhibit high druggability

**Figure 1:**
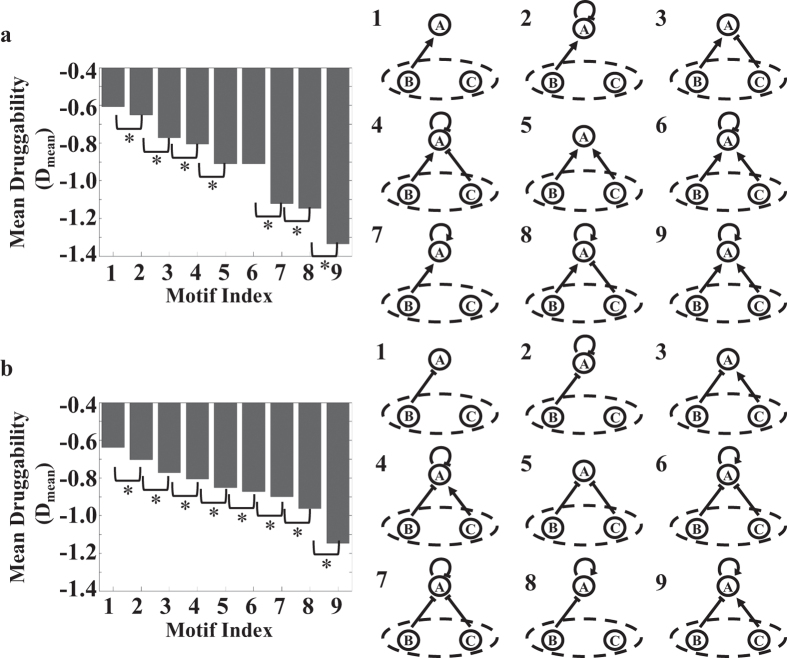
Effect of direct regulations on druggability. When detailed network motifs of a target are unknown, addition of a positive self-feedback loop to a drug target reduces druggability more than other regulations, unless it is counteracted by multiple negative direct regulations. Furthermore, addition of a direct regulation from buffer nodes with the same sign (positive indicates activation; negative indicates inhibition) as the pre-existing regulation reduces druggability more than the addition of a direct regulation from buffer nodes with the opposite sign. **(a)** Mean druggability (D_mean_) of representative motifs (consist of at least one positive direct regulation from buffer nodes). The motifs are grouped based on only the direct regulations to node A. (**b)** Mean druggability (D_mean_) of representative motifs (consist of at least one negative direct regulation from buffer nodes). The corresponding motifs are shown on the right of the bar graph. Asterisks indicate significant difference (N > 300,000, p-value < 0.05).

To start, we assume a scenario where we do not know about the detailed network motifs, but have the information on regulatory links that connect to node A. This scenario is common in drug target discovery because many *in vivo* biological interactions and networks remain unknown due to cells’ inherent complexity^24^. We grouped motifs that have the same number of positive and negative links to the target node and calculated the mean of their expected druggability (D_mean_). We separately sorted the groups that contain at least either one positive (Fig. 1a) or negative direct regulation (Fig. 1b) to the target. We find that D_mean_ drops when a self-feedback loop is added to a given motif (See Supplementary Information Text for steady-state analysis. Supplementary Figure S3). For example, starting from a single positive direct regulation (Fig. 1a, index 1), D_mean_ becomes more negative (less druggable) when a negative self-feedback loop is added to the network motifs (Fig. 1a, index 2). The addition of a positive self-feedback loop also reduces D_mean_ (Fig. 1a, index 7). Similarly, starting from a single negative direct regulation (Fig. 1b, index 1), D_mean_ becomes more negative (less druggable) when either a negative self-feedback loop (Fig. 1b, index 2) or positive self-feedback loop (Fig. 1b, index 8) is added to the network motifs. Furthermore, adding a positive self-feedback loop decreases druggability more drastically than a negative self-feedback loop (e.g. Fig. 1a, index 2 and 7; Fig. 1b, index 2 and 8). Our results suggest that positive self-feedback loop is stronger than negative self-feedback loop in countering drug inhibition unless it is weakened due to the presence of negative direct regulations (i.e. Fig. 1b, index 6 and 7. See Supplementary Information Text for steady-state analysis).

In addition, we find that D_mean_ drops when direct regulations are added to a given motif. For example, starting from a single direct regulation (Fig. 1a, index 1; Fig. 1b, index 1), D_mean_ becomes more negative (less druggable) when another positive direct regulation is added to the network motifs (Fig. 1a, index 5; Fig. 1b, index 3). Furthermore, the motifs with two direct regulations from buffer nodes of opposite signs (positive indicates activation; negative indicates inhibition) and either negative or no self-feedback loops (Fig. 1a, index 3 or 4; Fig. 1b, index 3 or 4) have higher druggability than the motifs that contain two direct regulations of the same signs (Fig. 1a, index 5 or 6; Figs 1b, 5 or 8 respectively). The results are explained by steady-state analysis (See Supplementary Information Text, Supplementary Figure S3 and Supplementary Table S1). Essentially, due to the mass conservation of the drug target, drug inhibition will lead to either stronger positive direct regulations or weaker negative direct regulations than without the drug. As a result, a higher number of direct regulations leads to weaker inhibitory effect of the drug and lower druggability of the target (See steady-state analysis and Supplementary Figure S3). Regulations with opposite signs compromise the tight control through counteraction of their impact on the drug target. We note that certain motifs seem to contradict the principles. Specifically, the addition of the second negative direct regulation increases D_mean_ when a positive self-feedback loop is present (Fig. 1b, index 6 and 8, or index 6 and 9). However, the results arise because the positive self-feedback loop, which has the strongest negative impact on druggability, is counteracted by multiple negative direct regulations (See Supplementary Information Text for magnitude analysis). In summary, when a target’s detailed network is unknown, we find that the addition of either positive or negative self-feedback loop reduces druggability. Yet, the addition of a positive self-feedback loop significantly reduces druggability unless it is counteracted by negative direct regulations. Furthermore, direct regulations with the same sign decrease druggability more than direct regulations with the opposite sign.

**Figure 2:**
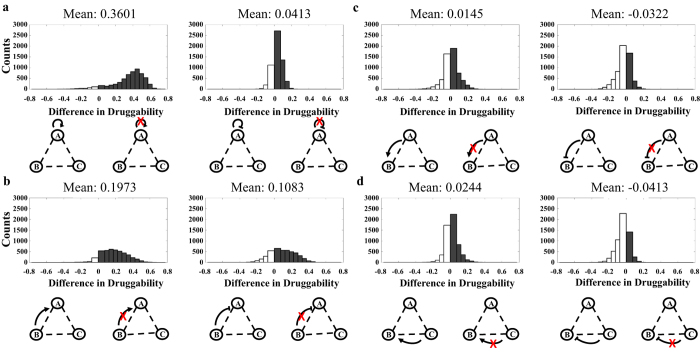
Effect of perturbation of single regulation on druggability. When detailed network motifs are considered, the perturbation of a positive self-feedback loop improves druggability of the target more effectively than the perturbation of other regulations (largest difference between perturbed and unperturbed motifs). Furthermore, perturbations of direct regulations from buffer nodes improve druggability of the target in general, but the perturbations of indirect regulations do not improve druggability. (**a)** Two motifs with (red cross) and without (no red cross) perturbation of a self-feedback loop are compared under identical background regulations. Perturbing only one regulation generates 3^8^ possible comparative pairs. Positive difference of the mean indicates improvement of druggability; negative difference of the mean indicates no improvement of druggability. Mean values of the histogram are calculated to evaluate the overall improvement in druggability. The perturbations of either the positive (left panel) or negative (right panel) self-feedback loop show improvement in druggability (positive mean values). Grey bars indicate cases where perturbation improves druggability. White bars indicate cases where perturbation does not improve druggability. (**b)** Two motifs with (red cross) and without (no red cross) perturbation of a direct regulation from buffer nodes are compared under identical background regulations. The perturbations of either the positive or negative direct regulation show improvement in druggability, which is indicated by positive mean values and larger area under grey bars than white bars. The improvement is less effective (less positive mean) than the improvement from the perturbation of the positive self-feedback loop (**a**). (**c**,**d)** Two motifs with (red cross) and without (no red cross) perturbation of an indirect regulation are compared under identical background regulations. The perturbations of indirect regulations generally do not improve druggability (mean values are all less than 0.03).

Next, we assume that the detailed network motifs are known, which emulates the scenario where sufficient omics data have been collected to form complete regulatory networks. We first investigated how perturbations of single regulation modulate druggability. To do this, we compared the druggability of motifs with and without a single regulation under identical background regulations (other regulations except the perturbed regulation). Essentially, we evaluated two motifs with the difference in only one regulatory link. We examined whether the removal of the regulation would improve (with positive difference in druggability in Fig. 2) or diminish (with negative difference in druggability in Fig. 2) druggability. We then quantified the number of motifs in each case (shown as histograms in Fig. 2). The mean value of the difference in druggability was calculated to evaluate the changes in druggability due to the perturbation. That is, a positive mean value implies that the perturbation of a certain regulation would improve druggability, whereas a negative mean value implies the opposite. Our results show that the removal of a positive self-feedback loop is more effective in improving druggability than the removal of a negative self-feedback loop, which is consistent with our conclusions from Fig. 1. Specifically, the removal of a positive self-feedback loop has a mean difference of 0.3601, whereas the removal of a negative self-feedback loop only demonstrates a mean difference of 0.0413 (Fig. 2a). Furthermore, the perturbation of direct regulations from buffer nodes generally improves druggability with mean difference of 0.1973 for a positive direct regulation and 0.1083 for a negative direct regulation (Fig. 2b). In contrast, the perturbations of indirect regulations do not improve druggability (mean difference that are less than 0.03, Fig. 2c,d).

**Figure 3:**
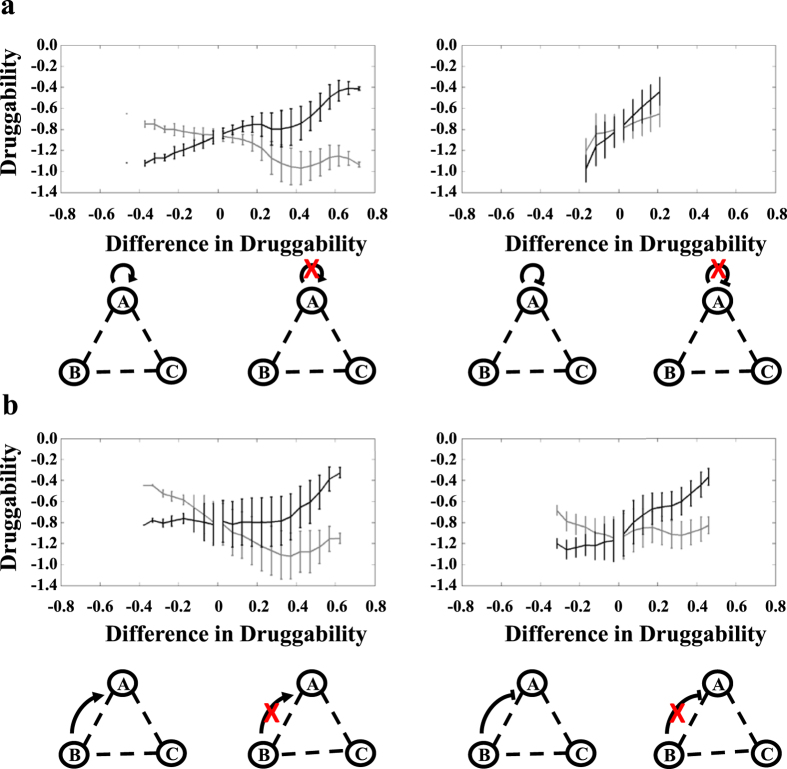
Improvement in druggability is dependent on the original values of druggability. (**a)** The perturbation of a positive self-feedback loop (left panel) improves the druggability from the lowest values to the highest values. In contrast, the perturbation of a negative self-feedback loop only improves druggability of the motifs with high original druggability. (**b**) Perturbations of direct regulations from buffer nodes show a wide range of variations. In general, the motifs with low druggability are improved by the perturbations. Two motifs with (red cross) and without (no red cross) perturbation of single regulation are compared under identical background regulations. For each comparison, the means of druggability for both motifs are calculated based on results from 1000 parameter sets. Both the means of druggability (y-axis) and difference in druggability (x-axis) are recorded. To better visualize the trends of the curves, results are grouped by 0.05 intervals in the x-axis on both sides of origin. Mean and standard derivation are calculated using data within each intervals. Black curves represent motifs with perturbation. Grey curves represent motifs without perturbation.

To further investigate how the perturbations of self-feedback loops and direct regulations from buffer nodes improve druggability, we compared the druggability of motifs with and without single regulation under identical background regulations by quantifying both their absolute druggability and changes in druggability (Supplementary Figure S4). We grouped the motifs with (grey curves in Fig. 3) or without (black curves in Fig. 3) a regulation and calculated their mean absolute druggability. The trends of the paired black and grey curves indeed reveal insights into the impact of the perturbations on druggability. For example, the perturbation of a positive self-feedback loop results in an increased curve (positive slope) for perturbed motifs and a decreased curve (negative slope) for unperturbed motifs (Fig. 3a, left panel). This trend shows that the motifs with higher improvement in druggability have worse druggability originally. Essentially, the perturbation inverts the druggability of the motif from the lowest to the highest. In contrast, the perturbation of a negative self-feedback loop results in increased curves for both perturbed and unperturbed motifs (Fig. 3a, right panel), which imply that the motifs with improved druggability already have high druggability originally. The perturbation of positive direct regulations from buffer nodes leads to a flat curve for perturbed motifs and a decreased curve for unperturbed motifs (between −0.4 to 0.4 in x-axis in Fig. 3b, left panel). The improvement in druggability is more significant if the motifs have lower druggability originally (druggability <−1.2 in Fig. 3b, left panel). The perturbation of a negative direct regulation from buffer nodes demonstrates an increased curve for perturbed motifs and a flat curve for unperturbed motifs (Fig. 3b, right panel). In summary, perturbations of positive self-feedback loop and direct regulations from buffer nodes improve druggability when the original druggability is low.

### Identify consensus network motifs that exhibit either high or low druggability

**Figure 4:**
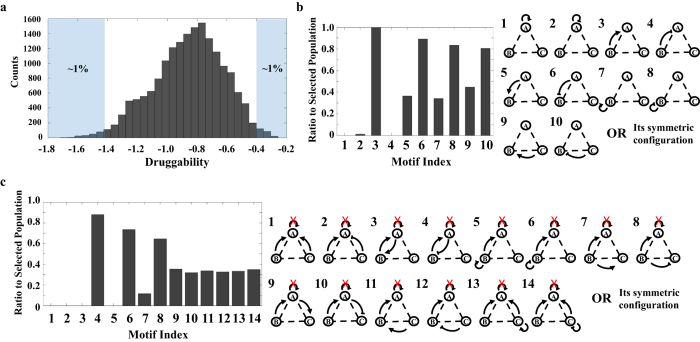
Consensus motifs that exhibit either high or low druggability. (**a)** Druggability of all possible motifs. Consensus motifs are determined by selecting the top or bottom 1% of the motifs (blue shaded regions). (**b)** Identify consensus motifs with high druggability. The selected top population is screened for consensus motifs through a two-step process. In step one, motifs are grouped by the presence of each of the nine regulations. Symmetric configurations of motifs are considered as identical and included in the same group. Only the regulations that appear more than 90% in the search are selected and used for the next step. The consensus motifs with high druggability contain at least one positive regulation from B (or C) to A (100%) and no self-feedback loops (0% for positive self-feedback loop and 1% for negative self-feedback loop). (**c)** In step two, we analyze all motifs that contain a positive regulation from B (or C) to A without self-feedback loops. The motifs that appear more than 85% are selected as consensus, druggable motifs. Thus, the consensus motif with high druggability does not have positive self-feedback loop, but contains a negative feedback loop, which consists of one positive regulation from B to A and one negative regulation from A to B. See Supplementary Figure S5 for the screening of consensus motifs with low druggability.

**Figure 5:**
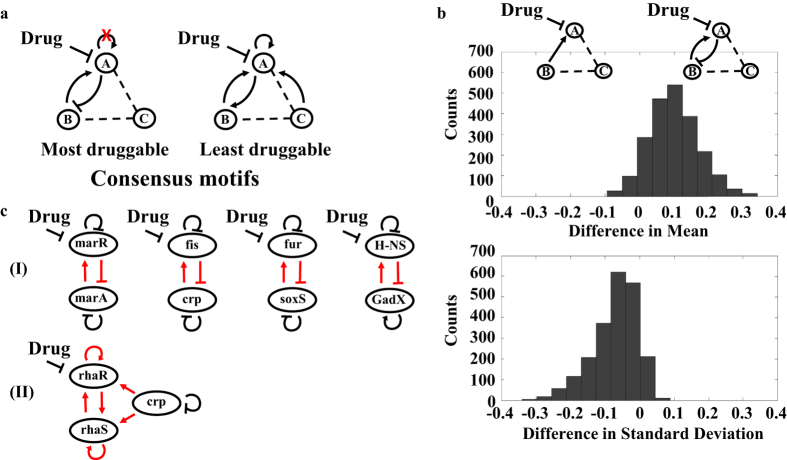
Consensus motifs that exhibit either high or low druggability exist in *E. coli* transcription networks. (**a)** The consensus motif with the highest druggability contains a negative feedback loop, which is formed by a positive regulation from node B (or C) to node A and a negative regulation from node A back to node B (or C). The most druggable, consensus motif cannot contain any positive self-feedback loop. In contrast, the consensus motif with the lowest druggability contains multiple positive direct regulations and one positive feedback loop. (**b)** The most druggable, consensus motif has a higher D_mean_ and lower standard derivation (SD) of D_mean_ when compared to the motifs without the negative feedback loop. A positive difference in either Mean or SD indicates that the motif with negative feedback loop has higher D_mean_ or SD than the one without negative feedback loop. A negative difference of Mean or SD indicates the opposite. (**c)** The transcription network of *E. coli* is screened for the presence of the consensus motifs. Four genes (panel I) have the same topology as the network motif with high druggability (**a**, left), and two genes (panel II) have the same topology as the network motif with low druggability (**a**, right).

Network motifs are known to underlie transcription networks, cellular signaling cascades, and neuronal networks. Ubiquitous network motifs with specific topology can be found in diverse organisms^25^. Therefore, we analyzed if consensus motifs exist with high or low druggability, which could lead to potential ways to predict druggable targets. We limited our search on motifs with non-isolated node A because important drug targets are often connected to a network^21^,^26^. We first ranked all motifs based on their druggability and selected the top 1% of the motifs to identify consensus motifs with high druggability (histogram in Fig. 4a). We note that changing the top percentage between 1% and 2% does not affect our conclusions. We first applied a coarse-grained search to identify the frequency of each regulation in the top population. We find that none of the motifs in the population have a positive self-feedback loop and only 1.14% of motifs contain a negative self-feedback loop. Furthermore, all motifs (100%) in the selected population contain one positive direct regulation (i.e. either from B to A or C to A) (Fig. 4b). Given this information, we next analyzed a subset of motifs that contains a positive direct regulation without a self-feedback loop. Specifically, we examined the frequency of all possible minimal combinations of regulations with the motifs. We find that 80% of the motifs contain a negative feedback loop without any self-feedback loop (Fig. 4c). Thus, the motif without positive self-feedback loop and with a negative feedback loop, which consists of one positive regulation from B to A and one negative regulation from A to B, is the consensus, high druggable motif (Fig. 5a). We note that all symmetrical motifs are considered in the analysis (e.g. negative feedback loops formed between either A and B, or A and C are considered identical). We also selected the bottom 1% of the population (Fig. 4a) to identify consensus motifs with low druggability. We conducted the same coarse and fine-grained analysis (Supplementary Figure S5). The motif with the least druggability consists of a positive self-feedback loop, multiple positive direct regulations from buffer nodes, and a positive feedback loop (Fig. 5a). Our results suggest that consensus motifs indeed exist with high or low druggability.

The druggable, consensus motif consists of a negative feedback loop, which is known to stabilize basal signal state and limit maximum output^27^. Therefore, we hypothesized that a negative feedback loop can increase stability in the concentration of a drug target to variation of circuit parameters, giving rise to less variation of druggability among the same network motifs with different kinetic parameters. Along this line, we analyzed the means and standard derivations (SDs) of the druggability of the most druggable motifs. We compared the differences in either the mean or standard derivation of each motif with and without a negative feedback loop (Fig. 5b). Consistent with our expectation, we find that the druggable, consensus motifs exhibit high druggability and low variation of druggability (Fig. 5b). In contrast, the least druggable, consensus motifs exhibit low druggability and high variation of druggability (Supplementary Figure S6a). To further characterize the consensus motifs, we investigate the inhibition rate of each consensus motif. We defined a new metric (t_1/2_) to quantify the time when the concentration of the drug target is reduced to half of its maximum change after addition of the drug (Supplementary Figure S6b). Our results do not show any correlation between druggability and inhibition rates of the consensus motifs (Supplementary Figure S6c).

### Determine natural drug targets embedded within highly druggable network motifs

Next, we applied the principles to predict potential druggable targets in *Escherichia coli*. We note that our results apply to both enzymatic reactions and genetic regulatory networks (see Supplementary Information Text for model construction). We first screened the gene network of *E. coli* and discovered four genes that are regulated by the highly druggable, consensus motifs (Fig. 5c, panel I). We also found two genes (e.g. *rhaR* and *rhaS*) that are regulated by the least druggable, consensus motifs (Fig. 5c, panel II).

**Figure 6:**
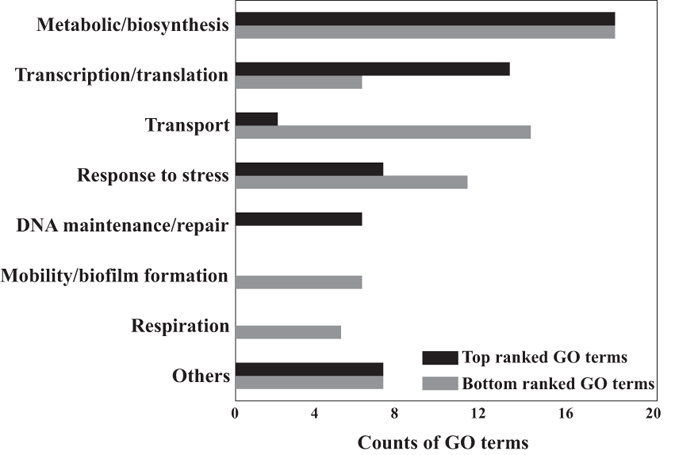
Biological functions of top and bottom ranked gene ontology (GO) terms. Based on the topology of network motifs, unique biological GO terms are predicted with high (top-ranked) or low (bottom-ranked) druggability. Both top- (black bars) and bottom-ranked (grey bars) GO terms include common functions, such as metabolic/biosynthesis, transcription/translation, transport, and response to stress associated functions. Unique functions, such as DNA maintenance/repair are identified in the top-ranked GO terms. Mobility/biofilm formation and respiration related functions are unique functions found in the bottom-ranked GO terms.

Our results reveal fundamental principles that could be used to analyze druggability of genes in natural networks. Therefore, we analyzed the gene networks of *E. coli* to reveal targets with high druggability, as well as their biological functions. To start, we ranked the druggability of all *E. coli* genes based on five criteria (See Methods for details). The five criteria are prioritized as follows: 1. The presence of the most druggable, consensus motifs (no positive self-feedback loop; more negative feedback loops, higher ranking); 2. The presence of the least druggable, consensus motifs (presence of positive self-feedback loop; more positive feedback loops, lower ranking); 3. Presence of a positive self-feedback loop with at least two more positive regulations than negative regulations (more positive over negative direct regulations from other transcription factors, lower ranking); 4. The number of direct regulations from other transcription factors (more direct regulations, lower ranking); 5. The signs of direct regulations from other transcription factors (opposite sign results in higher ranking than the same sign). Based on current knowledge of transcriptional regulatory network in *E. coli.*, we summarized the scores and ranking in Supplementary Information Datasets. To simplify our search, we selected 53 biological gene ontology (GO) terms that appeared in top ranking and 67 terms in bottom ranking. Both top and bottom GO terms contain transcription associated functions because the genes are involved in gene regulatory networks. In addition, we find GO terms related to metabolism, biosynthesis, transport, and response to stress in both top and bottom ranking. Among the top-ranked GO terms, we find unique biological functions that correspond to DNA maintenance and repair (Fig. 6 and Supplementary Information Datasets), including genes *ada, argR, lexA* and *pepA*. The bottom-ranked GO terms also include unique functions, such as mobility, biofilm formation and respiration, including genes *fumB, csgD, csgE, csgF, csgG, flhC, flhD, acnA, cyoA, cyoB, cyoC, cyoD, ndh, nirB*, and *nirD* (Fig. 6 & Supplementary Information Datasets). The results suggest a potential method to predict druggability of cellular targets based on network motifs. It can be applied to other organisms if their regulatory networks are known.

## Discussion

This work suggests that the network motif of a cellular target affects its druggability. We augment the definition of druggability as overall reduction of the drug-target upon drug addition. Our results support the literature data that binding affinity alone may not be sufficient to achieve effective treatment. The conclusions from this study can be used to identify potential cellular targets and then develop drug molecules that would have high specificity to the target. This work helps to close the gap between one-drug-one-target and network pharmacology paradigms by revealing fundamental topological features of a drug target that affect its druggability. We note that our findings are consistent with previous literature. For example, our conclusion with respect to direct regulations are consistent with previous findings on drug synergism. Through a computational simulation, combinatorial perturbations of parallel regulations towards a target are shown to enhance drug inhibition^28^. That is, perturbation of multiple direct regulations would improve druggability more than perturbation of a single regulation.

To enhance tractability of our models, we made a few assumptions in the mathematical models. First, we assume that the target and its regulatory nodes only change between active or inactive states without degradation. Second, the action of a drug is simulated by reducing the availability of the target, not degrading it. The above assumptions make sense because most drug molecules bind and deactivate the target’s functions^29^. Third, the change of drug concentration is chosen to be a step function. This assumption applies in the scenario when the drug is taken through intravenous infusions or the dosage is high and remains at saturation level during the period of treatment. We note that drug input profile has been mimicked with step and pulse functions. To examine the extension of our work to pulsatile drug input, we conducted another simulation using pulsatile drug input. The duration and amplitude of the pulsatile function were chosen so that the area under the curve remained unchanged from the original simulation within total simulation time (Supplementary Figure S7). We find that, with pulsatile drug input, consensus motifs with high or low druggability remain the same (Supplementary Figure S7). However, more thorough examination of pulsatile drug input is required in future work due to plausible emergent dynamics of such systems^30^,^31^. Fourth, we assume that the parameters in our model are not constrained and are randomly chosen from wide ranges (2 log-fold difference for catalytic rate constant, and 4 log-fold difference for half-maximum constant). We note that some factors, such as oxidation-reduction and molecular crowding, may influence the assumption. For instance, binding activity of NF-kappa B depends on its oxidation-reduction state^32^. The alteration of the binding activity may constrain the half-maximum constants in our model to either high or low values. Molecular crowding has been shown to limit diffusion rates and enhance binding rates of RNA polymerase to its promoter^33^. The decrease in diffusion rates may constrain catalytic rate constants in our model. However, we note that our conclusions should remain unchanged because they are not sensitive to specific parameter sets. Finally, we note that our conclusions will apply to networks that operate close to our model assumptions, in which the concentration of each variable is conserved and the regulatory links between each node do not exhibit cooperativity.

Our framework could be applied to the analysis of mammalian cells after the resolution of a few common challenges. First, metabolic networks^14^,^34^, protein-protein interaction networks^35^,^36^, and genetic regulatory networks^37^ of mammalian cells still have considerable gaps that may affect the fidelity of our analysis^38^. Furthermore, for complex cellular networks, we may need to cluster multiple nodes into a single effective node when applying our approach. Detecting and clustering the effective node from a highly connected interactome map remains non-trivial^39^,^40^. Second, a network motif of mammalian cells may be altered by other factors, such as DNA methylation^41^ and histone modification^42^ through gene silencing, which are not considered in our work.

To move this work forward, experimental validation of the basic principles will need to be conducted by inhibiting the predicted druggable targets in *E. coli.* The validated principles could serve as an additional selection criteria in current drug development, which could predict druggable new targets and reduce high attrition rates in one-drug-one-target strategy.

## Methods

### Three node motifs and model equations

Three node motifs have been used to simulate cellular networks^20^. We considered node A as the drug target that was inhibited by input I, and tracked the dynamics of node A (output). Node B and C served as buffer nodes that regulated node A. There were nine regulatory links. Each link represented one of the three possible regulations: negative, null, or positive. We considered all possible regulatory combinations with nine links (3^9^ = 19683 motifs) in our study.

We used Michaelis-Menten and ordinary differential equations to describe the regulatory links following a previous study^20^. Briefly, node A, B, and C represented enzymes that have active and inactive forms. The conversion between active and inactive forms were determined by the types of regulation. Specifically, the activation of A by B (positive regulation) was described by 
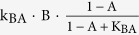
. The reaction dynamic of this type depended on the concentrations of the active regulator (B), inactive substrate (1-A), and two reaction parameters (catalytic rate constant, k_BA_ and half-maximum constant, K_BA_). Similarly, the deactivation of A by B (negative regulation) was described by 
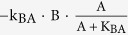
. The reaction rates depended on active substrate concentration (A). Furthermore, basal activation or deactivation was included in the model if a node was only regulated by either a negative or positive regulation respectively. The concentrations of regulatory basal enzymes (e.g. E_A_ for basal activating enzyme to A, F_A_ for basal deactivating enzyme to A) were set to be 0.1. When multiple regulations were acting on the same node, we assumed the effect to be additive. The ODEs of the model are shown in equation (1).


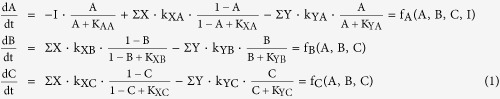


where A, B and C represent normalized concentrations ranging from 0 to 1, I represents the input, X represents the concentration of activating enzymes (A, B, C, E_A_, E_B_, or E_C_), Y represents the concentration of deactivating enzymes (A, B, C, F_A_, F_B_, or F_C_). k_ij_ and K_ij_ represent reaction parameters corresponding to regulator i to effector j.

### Data generation

To start the simulation, we set input to 0.1 and allowed the system to reach the first steady state (A_1_) with simulation time of t_1_=100. The input was then increased to 1.0 to simulate drug addition and the second concentration of the target (A_2_) was recorded at simulation time of t_2_ = 10. For each possible motif, 1000 parameter sets were randomized within the ranges of k ~ 0.1–10 with unit of one over time and K ~ 0.01–100 with unit of concentration. To evaluate the quantitative performance of the motif, we defined the druggability as 
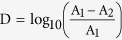
. Furthermore, we note that initial conditions alone are not sufficient to estimate druggability (i.e. sectioning along druggability in Supplementary Figure S8 does not show obvious separation of initial conditions).

### Statistical test

All statistical tests were performed with one-tailed unpaired two-sample t-test with 95% confidence intervals.

### Applications of generalized principles to *E. coli* genetic regulatory network

A database of transcriptional networks in *E. coli* was obtained from Ecocyc and analyzed in Matlab. The database includes regulations of individual gene by its transcription factors (TF) (1 represents up-regulation, −1 represents down-regulation, and 0 represents no regulation). Before analysis, genes without any regulations (isolated targets) were eliminated from the database. The consensus motifs with high or low druggability were defined in Matlab and searched for their presence in the *E. coli* genetic regulatory network.

To rank druggability of *E. coli* genes based on previously described criteria, we first determined several features of the network around each gene. Specifically, we recorded the number of most druggable, consensus negative feedback loops (score 1 in Supplementary Information Datasets), the number of least druggable, consensus positive feedback loops (score 2), the presence of a positive self-feedback loop (score 3) and a summation of direct regulations by other TFs of positive two or more (i.e. one positive and one negative direct regulations would have a summation of zero) (score 4), the number of direct regulations by other TFs (score 5), and the summation of direct regulations by other TFs (score 6). The scores were sorted with priorities from 1 to 6 with either descending (score 1) or ascending order (score 2–6). The biological gene ontology (GO) terms were downloaded from Gene Ontology Consortium. The biological GO terms were matched to specific genes from the TF database by their index. Top 53 and bottom 67 ranked GO terms were selected to include the same number of genes (39 genes). GO terms were then classified into functional groups, including metabolic/biosynthesis, transcription/translation, transport, response to stress, DNA maintenance/repair, mobility/biofilm formation, respiration, and others (see Supplementary Information Datasets).

## Additional Information

**How to cite this article**: Wu, F. *et al*. Network motifs modulate druggability of cellular targets. *Sci. Rep.* **6**, 36626; doi: 10.1038/srep36626 (2016).

**Publisher’s note:** Springer Nature remains neutral with regard to jurisdictional claims in published maps and institutional affiliations.

## Supplementary Material

Supplementary Information

Supplementary Information
