# Supplementary material for: Network motifs modulate druggability of cellular targets
Source: Sci Rep. 2016 Nov 8;6:36626. doi: 10.1038/srep36626 (PMC5100546; doi:10.1038/srep36626)
Supplement: Supplementary Information [file srep36626-s1.pdf]

Title: Network motifs modulate druggability of cellular targets

Author list: Fan Wu[1], Cong Ma[2] and Cheemeng Tan[1*]

Affiliations: [1] Department of Biomedical Engineering, University of California, Davis, 1 Shields Ave, Davis, CA 95616, USA and [2] Computational Biology Department, School of Computer Science, Carnegie Mellon University, 5000 Forbes Ave., Pittsburgh, PA 15213, USA

***To whom correspondence should be addressed. Email: cmtan@ucdavis.edu.**

Supplementary Information Text

Derivation of equation for steady-state analysis

To simplify our analysis, we used first-order Taylor series expansion of equation S1 around the first steady-state (s.s.) (A_s, B_s, C_s):

$$f_A(A,B,C,I) = \frac{d(A-A_s)}{dt} = f_A(A_s,B_s,C_s,I_1) + (A-A_s)\frac{\partial f_A}{\partial A} + (B-B_s)\frac{\partial f_A}{\partial B} + (C-C_s)\frac{\partial f_A}{\partial C} + (I-I_1)\frac{\partial f_A}{\partial I}$$

$$f_B(A,B,C) = \frac{d(B-B_s)}{dt} = f_B(A_s,B_s,C_s) + (A-A_s)\frac{\partial f_B}{\partial A} + (B-B_s)\frac{\partial f_B}{\partial B} + (C-C_s)\frac{\partial f_B}{\partial C} \qquad \text{(S1)}$$

$$f_C(A,B,C) = \frac{d(C-C_s)}{dt} = f_C(A_s,B_s,C_s) + (A-A_s)\frac{\partial f_C}{\partial A} + (B-B_s)\frac{\partial f_C}{\partial B} + (C-C_s)\frac{\partial f_C}{\partial C}$$

Since $f_A(A_s,B_s,C_s,I_1)$, $f_B(A_s,B_s,C_s)$ and $f_C(A_s,B_s,C_s)$ are all zeros, the system of ODEs becomes:

$$\frac{d\Delta A}{dt} = \Delta A\frac{\partial f_A}{\partial A} + \Delta B\frac{\partial f_A}{\partial B} + \Delta C\frac{\partial f_A}{\partial C} + \Delta I_1\frac{\partial f_A}{\partial I}$$

$$\frac{d\Delta B}{dt} = \Delta A\frac{\partial f_B}{\partial A} + \Delta B\frac{\partial f_B}{\partial B} + \Delta C\frac{\partial f_B}{\partial C}$$

$$\frac{d\Delta C}{dt} = \Delta A \frac{\partial f_C}{\partial A} + \Delta B \frac{\partial f_C}{\partial B} + \Delta C \frac{\partial f_C}{\partial C}$$

where $\Delta A = (A - A_s)$, $\Delta B = (B - B_s)$, $\Delta C = (C - C_s)$, and $\Delta I_1 = (I - I_1)$. We converted the system of ODEs into matrix form (equation S2).

$$\begin{bmatrix} \frac{d\Delta A}{dt} \\ \frac{d\Delta B}{dt} \\ \frac{d\Delta C}{dt} \end{bmatrix} = \begin{bmatrix} \alpha_{AA} & \beta_{BA} & \beta_{CA} \\ \beta_{AB} & \alpha_{BB} & \beta_{CB} \\ \beta_{AC} & \beta_{BC} & \alpha_{CC} \end{bmatrix} \cdot \begin{bmatrix} \Delta A \\ \Delta B \\ \Delta C \end{bmatrix} + \begin{bmatrix} \frac{\partial f_A}{\partial I} \\ 0 \\ 0 \end{bmatrix} \cdot \Delta I_1 \qquad \text{(S2)}$$

where $\begin{bmatrix} \alpha_{AA} & \beta_{BA} & \beta_{CA} \\ \beta_{AB} & \alpha_{BB} & \beta_{CB} \\ \beta_{AC} & \beta_{BC} & \alpha_{CC} \end{bmatrix}$ is the Jacobian matrix (J) and $\alpha_{ii} = \frac{\partial f_i}{\partial i}$ and $\beta_{ij} = \frac{\partial f_j}{\partial i}$.

To simplify our analysis, we assumed that the solutions applied to large perturbations between I_2 and I_1. Thus, at the second steady-state (A_{ss}, B_{ss}, C_{ss}), the equation S2 becomes

$$\begin{bmatrix} \frac{d(A_{ss}-A_s)}{dt} \\ \frac{d(B_{ss}-B_s)}{dt} \\ \frac{d(C_{ss}-C_s)}{dt} \end{bmatrix} = \begin{bmatrix} \alpha_{AA} & \beta_{BA} & \beta_{CA} \\ \beta_{AB} & \alpha_{BB} & \beta_{CB} \\ \beta_{AC} & \beta_{BC} & \alpha_{CC} \end{bmatrix} \cdot \begin{bmatrix} A_{ss} - A_s \\ B_{ss} - B_s \\ C_{ss} - C_s \end{bmatrix} + \begin{bmatrix} \frac{\partial f_A}{\partial I} \\ 0 \\ 0 \end{bmatrix} \cdot (I_2 - I_1) = 0 \qquad \text{(S3)}$$

We simplified the equations using $\begin{bmatrix} \Delta A_{s.s.} \\ \Delta B_{s.s.} \\ \Delta C_{s.s.} \end{bmatrix} = \begin{bmatrix} A_{ss} - A_s \\ B_{ss} - B_s \\ C_{ss} - C_s \end{bmatrix}$ and $\Delta I = (I_2 - I_1)$ to obtain

$$\begin{bmatrix} \alpha_{AA} & \beta_{BA} & \beta_{CA} \\ \beta_{AB} & \alpha_{BB} & \beta_{CB} \\ \beta_{AC} & \beta_{BC} & \alpha_{CC} \end{bmatrix} \cdot \begin{bmatrix} \Delta A_{s.s.} \\ \Delta B_{s.s.} \\ \Delta C_{s.s.} \end{bmatrix} + \begin{bmatrix} \frac{\partial f_A}{\partial I} \\ 0 \\ 0 \end{bmatrix} \cdot \Delta I = 0 \qquad \text{(S4)}$$

We solved for $\begin{bmatrix} \Delta A_{s.s.} \\ \Delta B_{s.s.} \\ \Delta C_{s.s.} \end{bmatrix}$ to obtain equation S5.

$$\begin{bmatrix}\Delta A_{s.s.}\\ \Delta B_{s.s.}\\ \Delta C_{s.s.}\end{bmatrix} = -J^{-1} \cdot \begin{bmatrix}\frac{\partial f_A}{\partial I}\\ 0\\ 0\end{bmatrix} \cdot \Delta I \qquad \text{(S5)}$$

where J is Jacobian matrix, which is $\begin{bmatrix}\alpha_{AA} & \beta_{BA} & \beta_{CA}\\ \beta_{AB} & \alpha_{BB} & \beta_{CB}\\ \beta_{AC} & \beta_{BC} & \alpha_{CC}\end{bmatrix}$.

Next, we solved for $\Delta A_{s.s.}$

$$\frac{\Delta A_{s.s.}}{\Delta I} = -{J_{11}}^{-1} \cdot \frac{\partial f_A}{\partial I} = -\left(\begin{bmatrix}\alpha_{AA} & \beta_{BA} & \beta_{CA}\\ \beta_{AB} & \alpha_{BB} & \beta_{CB}\\ \beta_{AC} & \beta_{BC} & \alpha_{CC}\end{bmatrix}^{-1}\right)_{11} \cdot \frac{\partial f_A}{\partial I}$$

Where $\left(\begin{bmatrix}\alpha_{AA} & \beta_{BA} & \beta_{CA}\\ \beta_{AB} & \alpha_{BB} & \beta_{CB}\\ \beta_{AC} & \beta_{BC} & \alpha_{CC}\end{bmatrix}^{-1}\right)_{11} = \frac{M_{11}}{|J|} = \frac{\begin{vmatrix}\alpha_{BB} & \beta_{CB}\\ \beta_{BC} & \alpha_{CC}\end{vmatrix}}{\begin{vmatrix}\alpha_{AA} & \beta_{BA} & \beta_{CA}\\ \beta_{AB} & \alpha_{BB} & \beta_{CB}\\ \beta_{AC} & \beta_{BC} & \alpha_{CC}\end{vmatrix}} =$

$\frac{\alpha_{BB}\alpha_{CC}-\beta_{CB}\beta_{BC}}{\alpha_{AA}\alpha_{BB}\alpha_{CC}+\beta_{BA}\beta_{AC}\beta_{CB}+\beta_{CA}\beta_{AB}\beta_{BC}-\alpha_{AA}\beta_{CB}\beta_{BC}-\alpha_{BB}\beta_{CA}\beta_{AC}-\alpha_{CC}\beta_{AB}\beta_{BA}}$, and M_{11} is the (1,1) minor of the Jacobian matrix.

As a result, we obtained equation S6

$$\frac{\Delta A_{s.s.}}{\Delta I} = -\frac{\partial f_A}{\partial I} \cdot \frac{\alpha_{BB}\alpha_{CC}-\beta_{CB}\beta_{BC}}{\alpha_{AA}\alpha_{BB}\alpha_{CC}+\beta_{BA}\beta_{AC}\beta_{CB}+\beta_{CA}\beta_{AB}\beta_{BC}-\alpha_{AA}\beta_{CB}\beta_{BC}-\alpha_{BB}\beta_{CA}\beta_{AC}-\alpha_{CC}\beta_{AB}\beta_{BA}} \qquad \text{(S6)}$$

For two-node cases, equation S5 was simplified to equation S7.

$$\begin{bmatrix}\Delta A_{s.s.}\\ \Delta B_{s.s.}\end{bmatrix} = -J^{-1} \cdot \begin{bmatrix}\frac{\partial f_A}{\partial I}\\ 0\end{bmatrix} \cdot \Delta I \qquad \text{(S7)}$$

where J in this case is $\begin{bmatrix}\alpha_{AA} & \beta_{BA}\\ \beta_{AB} & \alpha_{BB}\end{bmatrix}$. Solving for $\Delta A_{s.s.}$ yielded equation S8.

$$\frac{\Delta A_{s.s.}}{\Delta I} = -\frac{\partial f_A}{\partial I} \cdot \frac{\alpha_{BB}}{\alpha_{AA}\alpha_{BB}-\beta_{AB}\beta_{BA}} \qquad \text{(S8)}$$

For the motifs that do not have regulations to/from B and C, such as the examples shown in Supplementary Figure S3, most of the β_{ij} are zeros, except β_{BA} or β_{CA}. As a result, equation S6 and S8 become $\frac{\Delta A_{s.s.}}{\Delta I} = -\frac{\partial f_A}{\partial I} \cdot \frac{1}{\alpha_{AA}}$ after this simplification.

Steady-state analysis explains the computational results

To understand our findings from the computational studies, we performed steady-state analysis of the motifs (Supplementary Figure S3 and Supplementary Information Text). For the selected motifs, we approximated the relationship between changes of a drug target and a drug (equation S9).

$$\frac{\Delta A_{s.s.}}{\Delta I} = -\frac{\partial f_A}{\partial I} \cdot \frac{1}{\alpha_{AA}} \tag{S9}$$

where $\Delta A_{s.s.}$ is the change of drug target upon an input change (ΔI) (See previous section for derivation). A larger negative $\Delta A_{s.s}$ indicates a higher inhibition of A by I. Since ΔI is positive and constant, we expect that the motifs with high druggability should have more negative right-hand-side of equation S9. Furthermore, $-\frac{\partial f_A}{\partial I}$ is always positive and does not depend on node B or C since drug input only directly affects A. As a result, we focus our analysis on α_{AA}. Supplementary Figure S3 lists the α_{AA} terms of a few motifs. Motif I consists of only one positive direct regulation from B to A, whereas motif II consists of two positive direct regulations. Motif III consists of one positive and one negative direct regulations from B and C to A. Motif IV includes two negative direct regulations (Supplementary Figure S3). We find that α_{AA} has an extra term (second terms in motifs II and III from Supplementary Figure S3) when the motifs have more direct regulations (comparison between motifs I, II and III). The extra term

after the subtraction sign is always positive since all parameters and variables are positive, which decreases α_{AA} (become more negative) and thus increases (become less negative) the right-hand-side of equation S9. Therefore, more direct regulations from buffer nodes will make the motifs less druggable.

Next, we examine motifs II, III and IV to compare the effect of positive and negative direct regulations. Since α_{AA} is evaluated around the first steady-state after Taylor series expansion, we simplify each α_{AA} based on a magnitude analysis. We assume that the concentration of A at the first steady-state is large since it has two positive direct regulations, which means (1-A) is negligible. The α_{AA} of motif II is simplified to equation S10.

$$\alpha_{AA,II} = -\frac{k_{BA}\cdot B}{K_{BA}} - \frac{k_{CA}\cdot C}{K_{CA}} - \frac{I\cdot K_{IA}}{(A+K_{IA})^2} \qquad \text{(S10)}$$

In contrast, we assume that the concentration of A at the first steady-state is low for motif IV since it has two negative direct regulations. This assumption leads to the simplification of the α_{AA} for motif IV (equation S11).

$$\alpha_{AA,IV} = -\frac{k_{BA}\cdot B}{K_{BA}} - \frac{k_{CA}\cdot C}{K_{CA}} - \frac{I\cdot K_{IA}}{(A+K_{IA})^2} \qquad \text{(S11)}$$

We note that equations S10 and S11 are the same after simplification. In contrast, neither assumption is appropriate for motif III, which leads to equation S12.

$$\alpha_{AA,III} = -\frac{k_{BA}\cdot K_{BA}\cdot B}{(1-A+K_{BA})^2} - \frac{k_{CA}\cdot K_{CA}\cdot C}{(A+K_{CA})^2} - \frac{I\cdot K_{IA}}{(A+K_{IA})^2} \qquad \text{(S12)}$$

We note that the magnitude of the first and second terms in equation S12 are smaller than the magnitude of the corresponding terms in equations S10 and S11, because of the extra (1-A) and A terms in the denominator. As a result, α_{AA} of motif III is less negative than α_{AA} of motif II and

motif IV. Therefore, the motifs with direct regulations of opposite roles from buffer nodes generally have higher druggability than the ones with direct regulations of the same roles.

Similar analysis can be applied to self-feedback loops. The motifs with a negative self-feedback loop have an additional term in α_{AA} of motif V compared to motif I (equation S13, Supplementary Figure S3).

$$\alpha_{AA,V} = -\frac{k_{AA}\cdot(A^2+2K_{AA}\cdot A)}{(A+K_{AA})^2} - \frac{k_{BA}\cdot K_{BA}\cdot B}{(1-A+K_{BA})^2} - \frac{I\cdot K_{IA}}{(A+K_{IA})^2} \quad \text{(S13)}$$

If we assume that the concentration of A of motif V at the first steady-state is small, the first term becomes negligible. Thus, the contribution of the negative self-feedback loop to druggability is negligible as well.

A positive self-feedback loop gives rise to a different α_{AA} term (equation S14, Supplementary Figure S3, motif VI):

$$\alpha_{AA,VI} = -\frac{k_{AA}\cdot[-1-K_{AA}+2(1+K_{AA})\cdot A-A^2]}{(1-A+K_{AA})^2} - \frac{k_{BA}\cdot K_{BA}\cdot B}{(1-A+K_{BA})^2} - \frac{I\cdot K_{IA}}{(A+K_{IA})^2} \quad \text{(S14)}$$

We note that the term $-\frac{k_{AA}\cdot[-1-K_{AA}+2(1+K_{AA})\cdot A-A^2]}{(1-A+K_{AA})^2}$ changes sign depending on the concentration of A. Specifically, when the concentration of A is large, equation S14 was simplified to equation S15.

$$\alpha_{AA,VI} = -\frac{k_{AA}}{K_{AA}} - \frac{k_{BA}\cdot B}{K_{BA}} - \frac{I\cdot K_{IA}}{(A+K_{IA})^2} \quad \text{(S15)}$$

We note that the term contributed by positive self-feedback loop ($-\frac{k_{AA}}{K_{AA}}$) is negative, which makes α_{AA} of motif VI more negative compared to α_{AA} of motif I and increases (becomes less negative) right-hand side of equation S9. Thus, the druggability generally decreases when a

positive self-feedback loop is added. Furthermore, the magnitude of the first term in equation S15 is larger than the second term, since the concentration of B is between 0 and 1. Therefore, addition of a positive self-feedback loop has stronger impact to the druggability than other regulations. However, when the concentration of A is small, for example when multiple negative direct regulations are present, equation S14 is simplified as equation S16.

$$\alpha_{AA,VI} = \frac{k_{AA}}{1+K_{AA}} - \frac{k_{BA} \cdot K_{BA} \cdot B}{(1+K_{BA})^2} - \frac{I \cdot K_{IA}}{(A+K_{IA})^2} \tag{S16}$$

We note that the extra term ($\frac{k_{AA}}{1+K_{AA}}$) from positive self-feedback loop is positive, which makes α_{AA} of motif VI less negative and decreases (becomes more negative) right-hand side of equation S9. Thus, the druggability increases with the presence of a positive self-feedback loop in this scenario. This dependence of the self-positive feedback loop on initial conditions of A gives rise to the exception in Figure 1b. We note that the magnitude analysis uses the initial conditions of A to approximate and simplify the equations, but our results are not sensitive to the initial conditions of A because the druggability metric is normalized to initial conditions of the systems.

Genetic regulatory model construction

To construct gene regulatory models, we assumed that the translation of protein A is linearly proportional to the concentration of its mRNA and the transcription of mRNA is governed by the amount of available promoters that regulate gene A. We assumed that the promoter is regulated by binding or unbinding of a regulator. That is, the activation and inhibition of the promoter by other transcription factors (TFs) require the binding of a free regulator to available promoter and removal of a bound regulator from the promoter. The system of ODEs is constructed as equation S17.

$$\frac{dA_{protein}}{dt} = k_{tranlate,A} \cdot A_{mRNA} - k_{dA,1} \cdot A_{protein}$$

$$\frac{dA_{mRNA}}{dt} = k_{transcript,A} \cdot A_{promoter} - k_{dA,2} \cdot A_{mRNA} \tag{S17}$$

$$\frac{dA_{promoter}}{dt} = k_{activation,X} \cdot \frac{X(1-A_{promoter})}{K_{a,A}+1-A_{promoter}} - k_{inhibition,Y} \cdot \frac{Y \cdot A_{promoter}}{K_{i,A}+A_{promoter}} - I \cdot k_{inhibition} \cdot \frac{A_{promoter}}{K_I+A_{promoter}}$$

where $k_{translate,\ A}$ is rate constant for translation, $k_{transcript,\ A}$ is rate constants for transcription, $k_{dA,1}$ is degradation constant for protein of A, and $k_{dA,2}$ is degradation constants for mRNA of A. X and Y are concentrations of activators and inhibitors. $k_{activation,X}$, $k_{inhibition,Y}$ are rate constants, and $K_{a,A}$, $K_{i,A}$ are half-max constants for activation and inhibition by transcription factor X and Y to the promoter of A respectively. The rate and degradation constants have unit of one over time, and the half-max constants have unit of concentration. The drug input I inhibits the promoter of A. We note that the activation by the TFs is determined by the amount of occupied promoter (1-$A_{promoter}$), whereas the inhibition is determined by the amount of available promoter ($A_{promoter}$). Next, we assumed QSSA on mRNA to obtain equation S18. That is,

$$\frac{dA_{mRNA}}{dt} = k_{transcript,A} \cdot A_{promoter} - k_{dA,2} \cdot A_{mRNA} = 0 \tag{S18}$$

This assumption led to equation S19.

$$A_{mRNA} = \frac{k_{transcript,A}}{k_{dA,2}} \cdot A_{promoter} \tag{S19}$$

We plugged-in equation S19 into equation S17 and obtained equation S20.

$$\frac{dA_{protein}}{dt} = \frac{k_{tranlate,A} \cdot k_{transcript,A}}{k_{dA,2}} \cdot A_{promoter} - k_{dA,1} \cdot A_{protein}$$

$$\frac{dA_{promoter}}{dt} = k_{activation,X} \cdot \frac{X(1-A_{promoter})}{K_{a,A}+1-A_{promoter}} - k_{inhibition,Y} \cdot \frac{Y \cdot A_{promoter}}{K_{i,A}+A_{promoter}} - I \cdot k_{inhibition} \cdot \frac{A_{promoter}}{K_I+A_{promoter}} \quad \text{(S20)}$$

We note that the concentration of protein A is only governed by the available amount of promoter and its degradation. Therefore, the concentration of $A_{protein}$ can be approximated by the value of $A_{promoter}$. Thus, equation S20 essentially has the same format as equation 1 in main text. Therefore, our conclusions are applicable to transcription networks as well.

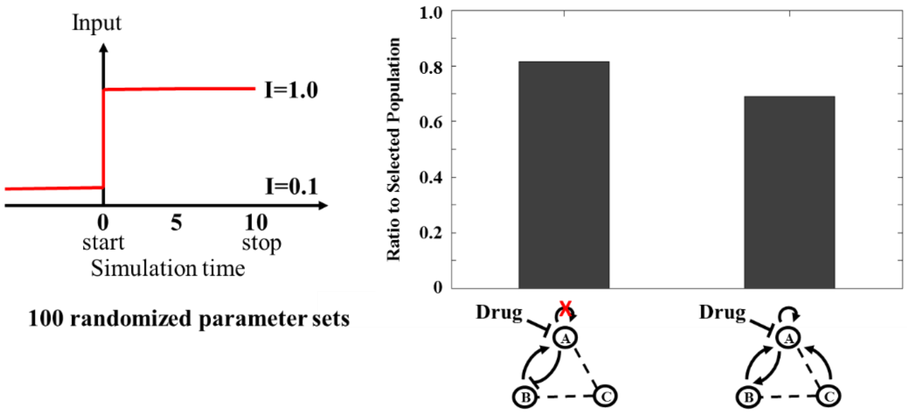

Supplementary Figure S1. Effect of sample size on the computational analysis. Our conclusions do not change with a different size of parameter set. A simulation with 100 randomized parameter sets was conducted and analyzed based on the same method (Figure 4). Consensus motifs with either high or low druggability remain unchanged when compared to the results in Figure 5. Bars represent the ratios of consensus motifs in top 1% (left) or bottom 1% (right) of all motifs.

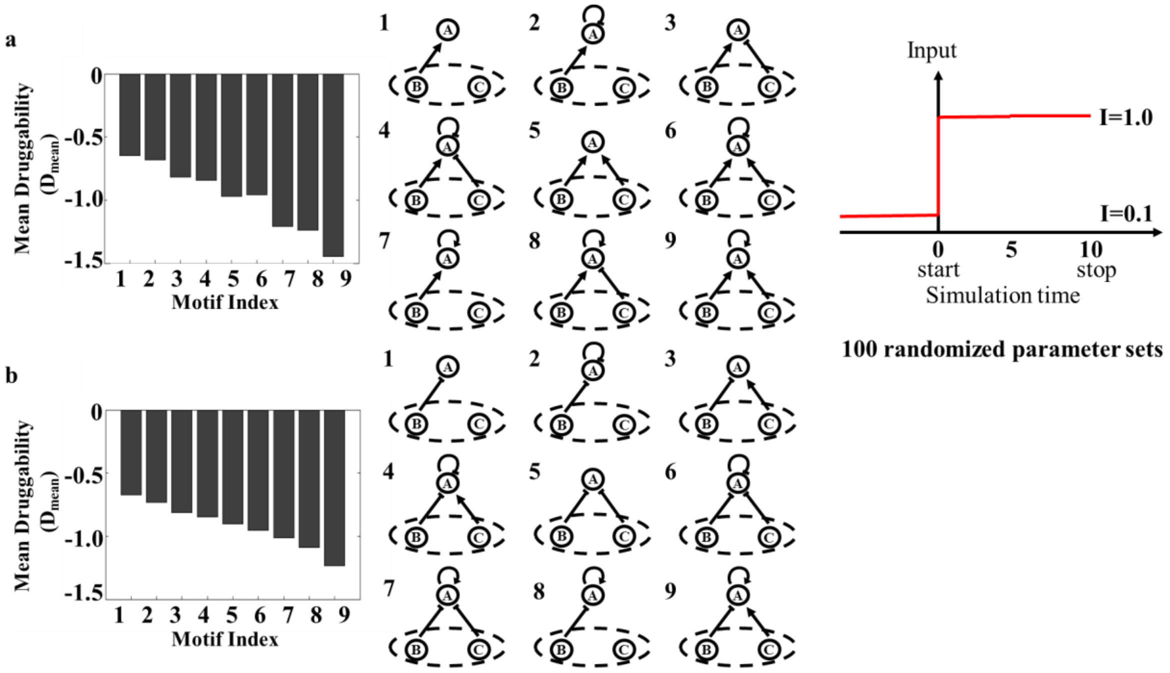

Supplementary Figure S2. Effect of parameter size on the computational analysis. Generalized fundamental principles are not sensitive to parameter size. A small size of randomized parameters (100 sets) is used for the simulation. Our conclusions from Figure 1 remain unchanged. Specifically, when detailed network motifs of a target are unknown, addition of a positive self-feedback loop to a drug target reduces druggability more than other regulations. Furthermore, addition of a direct regulation from buffer nodes with the same sign (positive indicates activation; negative indicates inhibition) to a pre-existing regulation reduces druggability more than the addition of a direct regulation with the opposite sign.

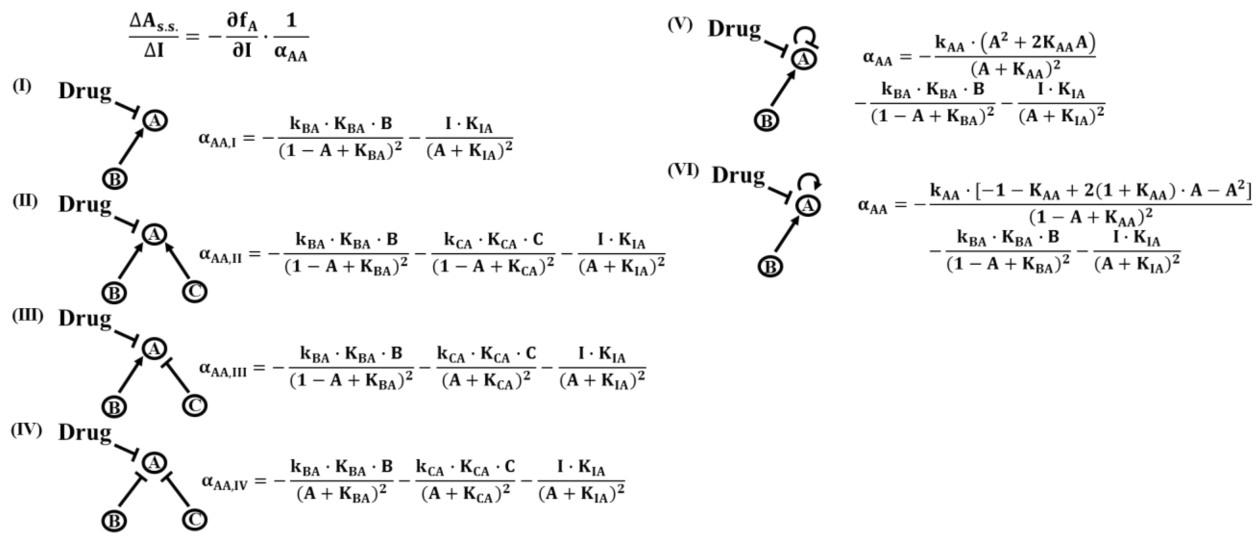

Supplementary Figure S3. Steady-state analysis of representative motifs. The relationship between concentration changes of a drug target and a drug is approximated using α_{AA} based on the equation $\frac{\Delta A_{s.s.}}{\Delta I} = -\frac{\partial f_A}{\partial I} \cdot \frac{1}{\alpha_{AA}}$. See Supplementary Information Text (equations S1-S16) for the detailed analysis of each motif. The motifs include one positive direct regulation from a buffer node (Motif I), two direct regulation of the same type (Motif II and IV), two direct regulation of the opposite type (Motif III), one negative self-feedback loop (Motif V), and one positive self-feedback loop (Motif VI).

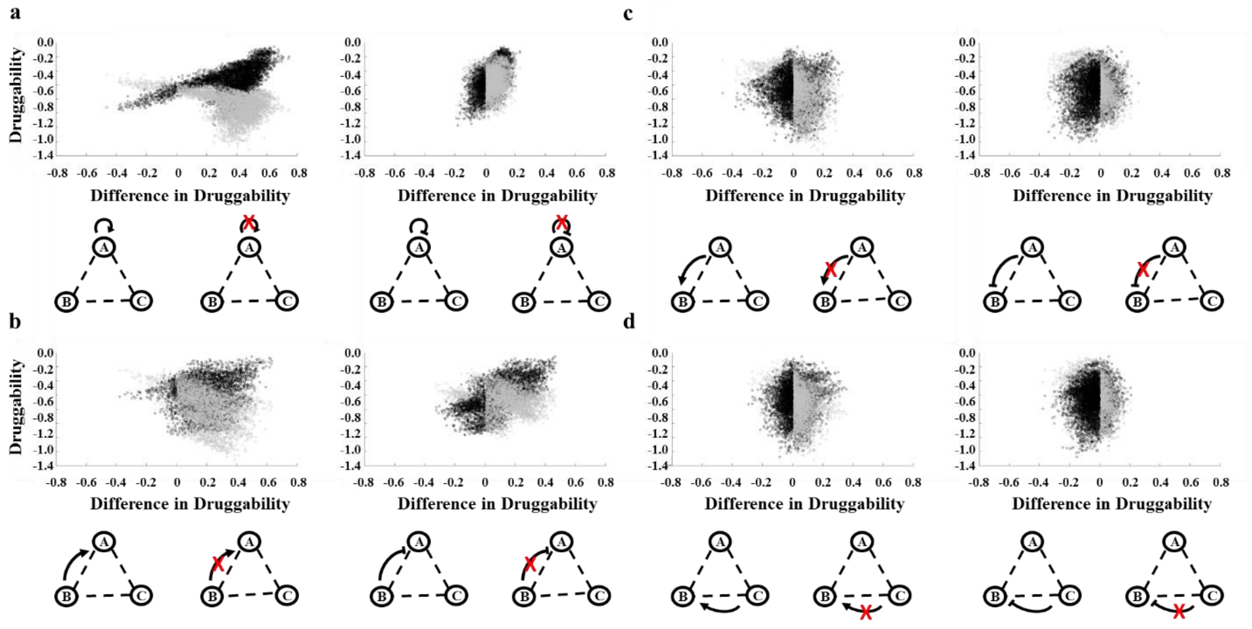

Supplementary Figure S4. Effect of perturbations of single regulation on druggability. Two motifs with (red cross) and without (no red cross) perturbation of single regulation are compared under identical background regulations. For each comparison, the means of druggability for both motifs are calculated based on results from 1000 parameter sets. Both the means of druggability (y-axis) and difference in druggability (x-axis) are recorded in scatter plots. Black dots represent the motifs with perturbation, whereas grey dots represent the motifs without perturbation. **a)** Perturbations of a positive or negative self-feedback loop show different trends. The trend of the scatter plot is simplified and evaluated in Figure 3a. **b)** Perturbations of direct regulations from buffer nodes show a wide range of variations. The trend of the scatter plot is simplified and evaluated in Figure 3b. **c&d)** Perturbations of indirect regulations do not show any obvious trends because their perturbations do not improve druggability.

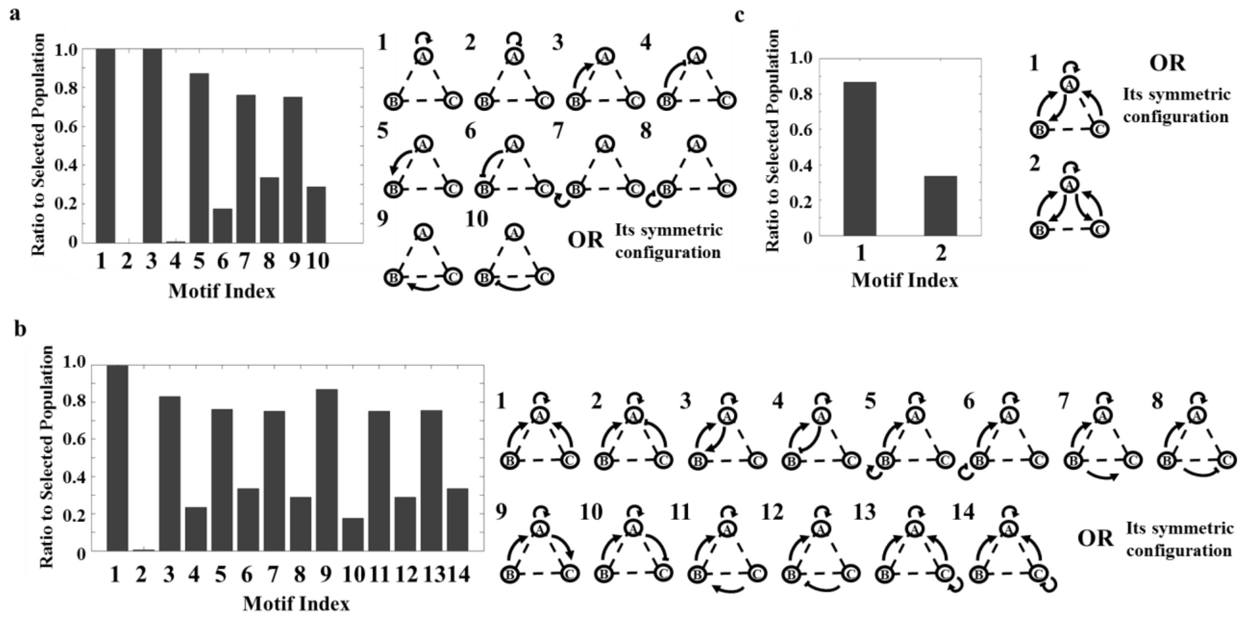

Supplementary Figure S5. Identify consensus motifs with low druggability. The selected bottom population is screened for consensus motifs through a three-step search. **a)** In step one, motifs are grouped by the presence of each of the nine regulations. Symmetric configurations of motifs are considered as identical and included in the same group. Only the regulations that appeared more than 90% in the search are selected and used in the next step. The consensus motifs with low druggability contain at least one positive regulation from B (or C) to A (100%) and a positive self-feedback loop (100%). **b)** In step two, we analyze the subset of motifs that contain a positive regulation from B (or C) to A and a positive self-feedback loop. Multiple combinations have more than 80% ratios. Therefore, we perform an extra step to reduce the number of consensus motifs. The motifs with higher than 90% ratios are selected for the next step. **c)** In step three, we find a motif with >85% ratio, which is selected as the consensus motif with low druggability. Thus, the consensus motifs with low druggability have a positive self-feedback loop, multiple positive direct regulations from buffer nodes, and a positive feedback loop.

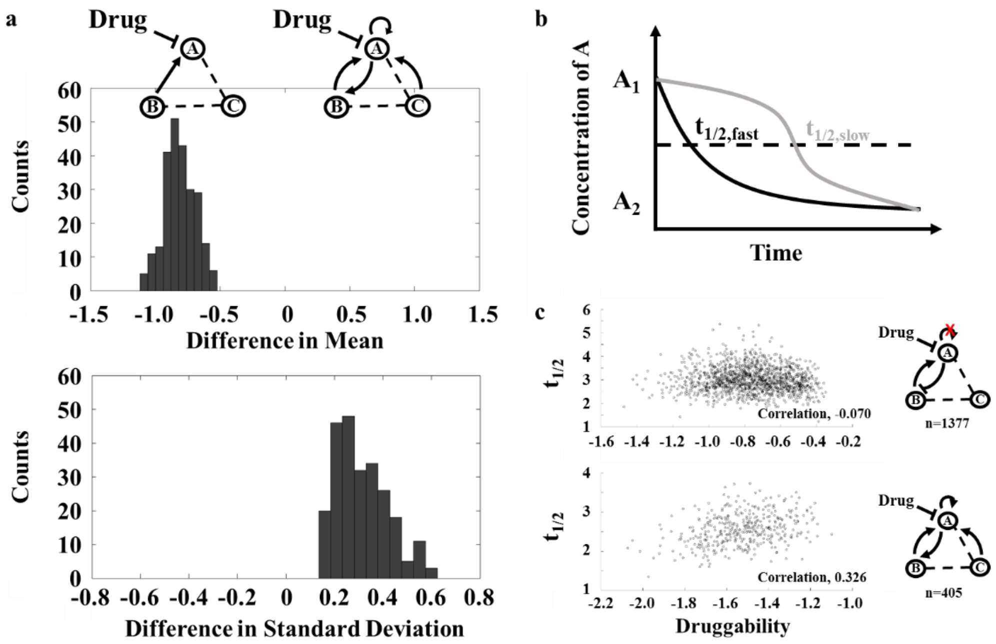

Supplementary Figure S6. Characteristics of the consensus motifs. a) The least druggable, consensus motif has a lower D_{mean} and higher standard derivation (SD) of D_{mean} when compared to the motif without multiple positive regulations, a positive self-feedback loop, or a positive feedback loop. A positive difference in either Mean or SD indicates that the least druggable, consensus motif has higher D_{mean} or SD than the one without multiple positive regulations, a positive self-feedback loop, or a positive feedback loop. A negative difference of Mean or SD indicates the opposite. **b)** To compare inhibition rates of consensus motifs, we calculate the time $t_{1/2}$ when the concentration of the drug target is inhibited to half of its maximum change. A drug target can exhibit either fast inhibition (black curve, smaller $t_{1/2,fast}$) or slow inhibition (grey curve, larger $t_{1/2,slow}$). **c)** Using the simulation with 100 parameter sets (Supplementary Figure S1 and S2), we select all consensus motifs with either high (n=1377) or low (n=405) druggability. The Pearson correlation coefficients are -0.070 for consensus motifs with high druggability and 0.326 for consensus motifs with low druggability ($p<0.01$). Based on the results, there is no

correlation between druggability and $t_{1/2}$ of the consensus motifs with either high or low druggability.

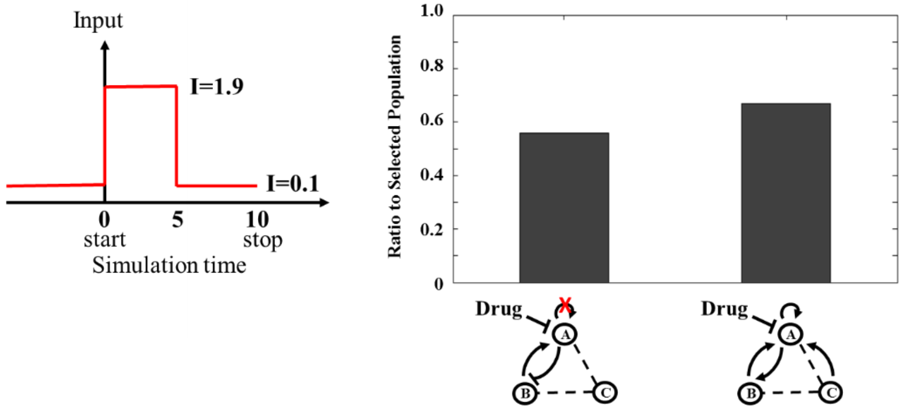

Supplementary Figure S7. Effect of pulsatile drug input on consensus motifs with high and low druggability. Consensus motifs with either high or low druggability remain the same with pulsatile drug input. The duration and amplitude of the pulse-function were chosen so that the area under the curve remain unchanged from the original simulation within total simulation time (t=10). Specifically, the input was 0.1 for $t<0$ and $t>5$, and changed to 1.9 when $0<t<5$. Through the simulation, we identified the same consensus motifs with either high or low druggability as the original conclusion (Figure 5a). The consensus motif with high druggability is present in 55% of the top druggable motifs (top 1% of all motifs, left bar), and the consensus motif with low druggability is present in 67% of the least druggable motifs (bottom 1% of all motifs, right bar).

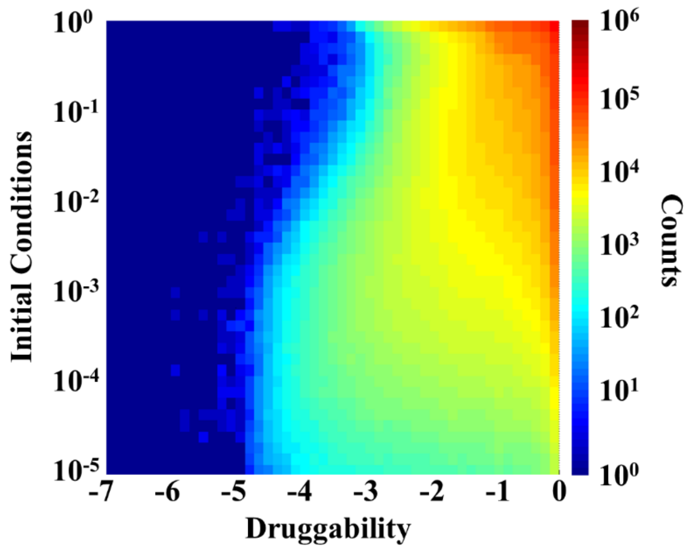

Supplementary Figure S8. Effect of initial condition on druggability. The initial condition of a drug target is not sufficient to predict druggability of the target. To investigate the correlation between initial conditions and druggability, we plot druggability versus the initial condition of all motifs, and summarize the counts in a heatmap. In the range of all initial conditions, the distribution of druggability is similar. Therefore, initial condition alone is not sufficient to determine a target's druggability.

	D_{mean}		D_{mean}		D_{mean}	Input	Conclusion
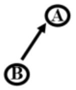	-0.6702	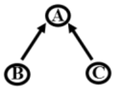	-1.0578	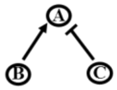	-0.9012	0.1→1	Self-positive regulation reduces the druggability more than other types of regulation. In contrast, self-negative regulation reduces the druggability less than other types of regulation.
	-0.1762		-0.3305		-0.3262	0.1→10	
	-0.6423		-1.0241		-0.8692	0.01→1	
		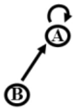	-1.1907	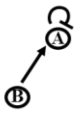	-0.7380	0.1→1	
			-0.3578		-0.2288	0.1→10	
			-1.1553		-0.7092	0.01→1	

	D_{mean}		D_{mean}		D_{mean}	Input	Conclusion
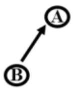	-0.6702	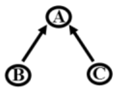	-1.0578	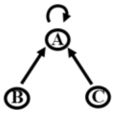	-1.3920	0.1→1	Increased number of direct regulations → druggability decreases.
	-0.1762		-0.3305		-0.4439	0.1→10	
	-0.6423		-1.0241		-1.3633	0.01→1	
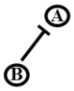	-0.7301	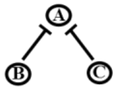	-0.9860	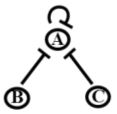	-1.0420	0.1→1	
	-0.2887		-0.4433		-0.4780	0.1→10	
	-0.6950		-0.9485		-1.0037	0.01→1	

	D_{mean}		D_{mean}		D_{mean}	Input	Conclusion
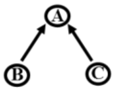	-1.0578	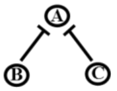	-0.9860	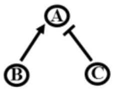	-0.9012	0.1→1	Non-self direct regulations with the opposite signs are more druggable than the ones with the same signs
	-0.3305		-0.4433		-0.3262	0.1→10	
	-1.0241		-0.9485		-0.8692	0.01→1	

Supplementary Table S1. Effect of drug input concentrations on the analysis of druggability. Concentrations of the drug input do not affect conclusions of this work. Simulations were conducted with different drug concentrations. D_{mean} of representative motifs were calculated.
